# Decoding the molecular landscape: A novel prognostic signature for uveal melanoma unveiled through programmed cell death-associated genes

**DOI:** 10.1097/MD.0000000000038021

**Published:** 2024-05-03

**Authors:** Zibin Liu, Lili Yu, Jian Lai, Rui Zhang

**Affiliations:** a Department of Ophthalmology, Hangzhou TCM Hospital Affiliated to Zhejiang Chinese Medical University, Hangzhou, Zhejiang, China; b Department of Pediatrics, Hangzhou Linping TCM Hospital, Hangzhou, Zhejiang, China.

**Keywords:** consensus clustering, immune infiltration, Lasso Cox regression analysis, programmed cell death, uveal melanoma

## Abstract

Uveal melanoma (UM) is a rare but aggressive malignant ocular tumor with a high metastatic potential and limited therapeutic options, currently lacking accurate prognostic predictors and effective individualized treatment strategies. Public databases were utilized to analyze the prognostic relevance of programmed cell death-related genes (PCDRGs) in UM transcriptomes and survival data. Consensus clustering and Lasso Cox regression analysis were performed for molecular subtyping and risk feature construction. The PCDRG-derived index (PCDI) was evaluated for its association with clinicopathological features, gene expression, drug sensitivity, and immune infiltration. A total of 369 prognostic PCDRGs were identified, which could cluster UM into 2 molecular subtypes with significant differences in prognosis and clinicopathological characteristics. Furthermore, a risk feature PCDI composed of 11 PCDRGs was constructed, capable of indicating prognosis in UM patients. Additionally, PCDI exhibited correlations with the sensitivity to 25 drugs and the infiltration of various immune cells. Enrichment analysis revealed that PCDI was associated with immune regulation-related biological processes and pathways. Finally, a nomogram for prognostic assessment of UM patients was developed based on PCDI and gender, demonstrating excellent performance. This study elucidated the potential value of PCDRGs in prognostic assessment for UM and developed a corresponding risk feature. However, further basic and clinical studies are warranted to validate the functions and mechanisms of PCDRGs in UM.

## 1. Introduction

Melanoma is a malignant tumor originating from melanocytes, characterized by high invasiveness and metastatic potential. Based on the site of occurrence, melanoma can be classified into cutaneous melanoma, mucosal melanoma, and uveal melanoma (UM), among others.^[1]^ UM is the most common primary intraocular tumor, although its incidence is relatively low, it has a poor prognosis, with an overall 5-year survival rate of only around 60%.^[2]^ Currently, high-risk UM patients are primarily treated with radical surgeries such as enucleation, but a considerable proportion of patients ultimately develop distant metastases, with a median overall survival of only 1 year.^[3]^ Therefore, accurately assessing the prognostic risk of UM patients and formulating individualized treatment strategies is crucial for improving their quality of life.

Programmed cell death (PCD) refers to the orderly process of cell death through intrinsic molecular mechanisms encoded by genes, playing a pivotal role in maintaining homeostasis, tissue development, and tumor progression.^[4]^ Increasing evidence suggests that aberrant expression of PCD-related genes is closely associated with tumor initiation, progression, and metastasis.^[5]^ For instance, dysregulation of the apoptosis-related BCL2 family members can lead to inappropriate survival of cancer cells^[6]^; deficiency in the mitochondrial quality control genes PINK1 and Parkin can cause mitochondrial dysfunction, promoting tumor progression^[7,8]^; activation of key molecules such as GSDMD and MLKL in the inflammatory PCD pathway can induce immunogenic cell death, eliciting antitumor immune responses.^[9,10]^ Therefore, in-depth exploration of molecular markers associated with PCD and their relationship with UM prognosis may provide new insights for individualized risk assessment and targeted therapy.

In this study, we employed systems biology approaches to analyze transcriptomic and clinicopathological data of UM from public databases, aiming to elucidate the potential value of PCD-related genes (PCDRGs) in UM prognosis prediction. Based on the prognostic PCDRGs, we performed molecular subtyping of UM and constructed a risk signature to quantify the risk of poor prognosis. Additionally, we systematically analyzed the potential roles of PCDRGs in drug sensitivity and the tumor immune microenvironment. This study contributes to elucidating the potential roles of PCDRGs in tumor development, targeted drug development, and guiding personalized treatment.

## 2. Materials and methods

### 2.1. Data collection

Transcriptomic data, somatic mutation data, and clinicopathological characteristics of 80 UM patients were obtained from The Cancer Genome Atlas (TCGA, https://portal.gdc.cancer.gov/) database (TCGA-UVM project, accessed date: February 10, 2024). The GSE84976 and GSE22138 datasets were retrieved from the Gene Expression Omnibus (https://www.ncbi.nlm.nih.gov/gds/) database as validation cohorts, comprising 28 and 63 UM cases, respectively. A list of 1560 PCD-related genes (PCDRGs) was obtained from previous studies^[11]^ (Table S1, Supplemental Digital Content, http://links.lww.com/MD/M354).

### 2.2. Molecular subtyping

Univariate Cox regression analysis was performed to identify PCDRGs significantly associated with UM prognosis (*P* < .05), and consensus clustering analysis was conducted using the ConsensusClusterPlus^[12]^ package. The partitioning around medoids clustering method and the “pearson” distance function were employed for clustering analysis, and differences in prognosis and clinicopathological features among subtypes were analyzed. Principal component analysis (PCA) was performed using the prognostic PCDRGs.

### 2.3. Construction of risk signature

PCDRGs with *P* < .005 in the univariate Cox regression analysis were selected, and the Least Absolute Shrinkage and Selection Operator Cox regression analysis was performed using the glmnet^[13]^ package to construct the PCDRG-derived index (PCDI), which was calculated as follows: PCDI = Σ(β_i_ × exp_i_), where β_i_ is the coefficient of gene i, and exp_i_ is the expression level of gene i. Survival analysis and PCA were conducted to evaluate the performance of PCDI in molecular subtyping within the TCGA-UVM cohort.

### 2.4. Somatic mutation analysis

The maftools^[14]^ package was used to analyze the somatic mutation characteristics of UM patients.

### 2.5. Drug sensitivity analysis

The pRRophetic^[15]^ package was employed to analyze the sensitivity to 45 drugs, using the pRRopheticPredict() function, and batch correction was performed using the ComBat method. The Wilcoxon test was used to evaluate the differences in drug sensitivity between the High_PCDI and Low_PCDI groups, and the correlation between PCDI and drug sensitivity was analyzed.

### 2.6. Immune infiltration analysis

The IOBR^[16]^ package, which includes algorithms such as CIBERSORT, EPIC, xCell, MCP-counter, ESTIMATE, TIMER, quanTIseq, and immune phenotype scores (IPS), was used to assess tumor immune cell infiltration in the TCGA-UVM cohort. The Wilcoxon test was employed to evaluate the differences between the High_PCDI and Low_PCDI groups.

### 2.7. Enrichment analysis

The edgeR^[17]^ package was used to analyze differentially expressed genes between the High_PCDI and Low_PCDI groups, with *P* = .05 and |log(fold change)| > 2 as the cutoff criteria. Enrichment analysis, including Gene Ontology and Kyoto Encyclopedia of Genes and Genomes enrichment analyses, was performed using the clusterProfiler^[18]^ package, with a significance threshold of 0.05.

### 2.8. Construction and evaluation of nomogram

Independent prognostic factors for UM were identified through multivariate Cox regression analysis and used to construct a nomogram. The rms package was employed for nomogram construction and visualization. The performance of the nomogram was evaluated using receiver operating characteristic curve analysis, calibration curve analysis, and decision curve analysis using the rmda package.

## 3. Results

### 3.1. Molecular subtyping of UM based on prognostic PCDRGs

**Figure 1.**
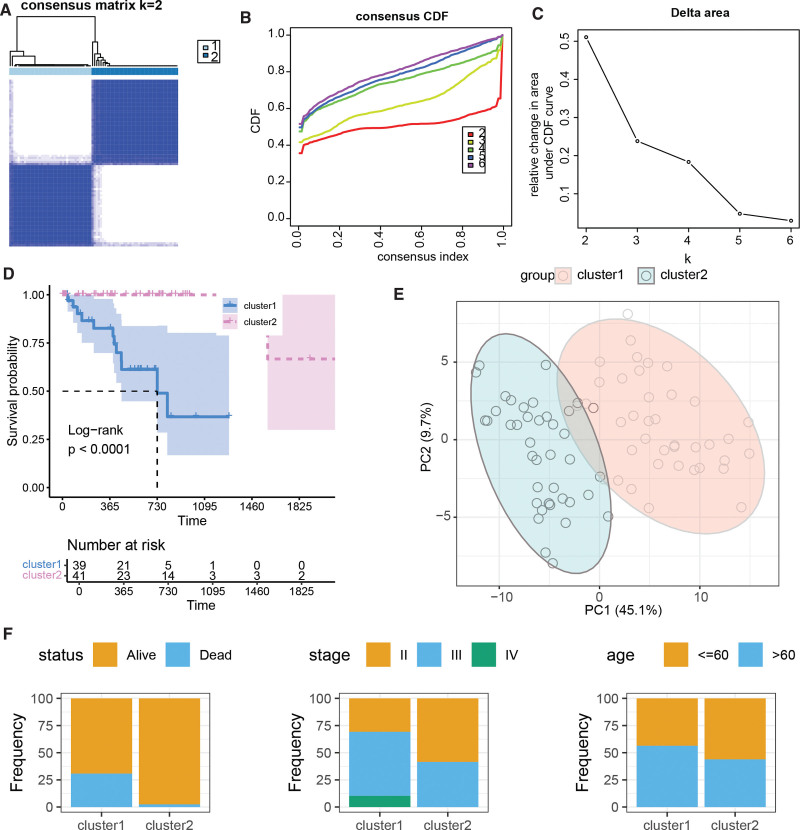
Molecular subtyping of the TCGA-UVM cohort based on prognostic PCDRGs. (A)–(C) Consensus clustering based on 369 prognostic PCDRGs. (D) Kaplan–Meier survival curves and log-rank test between cluster 1 and cluster 2 subtypes. (E) Principal component analysis based on prognostic PCDRGs. (F) Distribution of clinical pathological features, including status, stage, and age, between cluster 1 and cluster 2 subtypes. PCDRGs = programmed cell death-related genes, TCGA = The Cancer Genome Atlas.

Among the 1560 PCDRGs analyzed, 369 genes exhibited a significant association with UM prognosis (Table S2, Supplemental Digital Content, http://links.lww.com/MD/M355). Consensus clustering analysis based on these prognostic PCDRGs identified 2 distinct UM subtypes, designated as cluster 1 and cluster 2 (Fig. 1A–C). Survival analysis revealed a significantly worse prognosis for patients in cluster 1 compared to cluster 2 (*P* < .0001, Fig. 1D). PCA demonstrated clear separation between the 2 molecular subtypes (Fig. 1E), indicating that these prognostic PCDRGs can effectively stratify UM patients with favorable and unfavorable outcomes. Notably, cluster 1, associated with poorer prognosis, exhibited a higher proportion of stage III and IV UM patients compared to the better prognostic cluster 2 (Fig. 1F).

### 3.2. PCDRG-derived prognostic index (PCDI)

**Figure 2.**
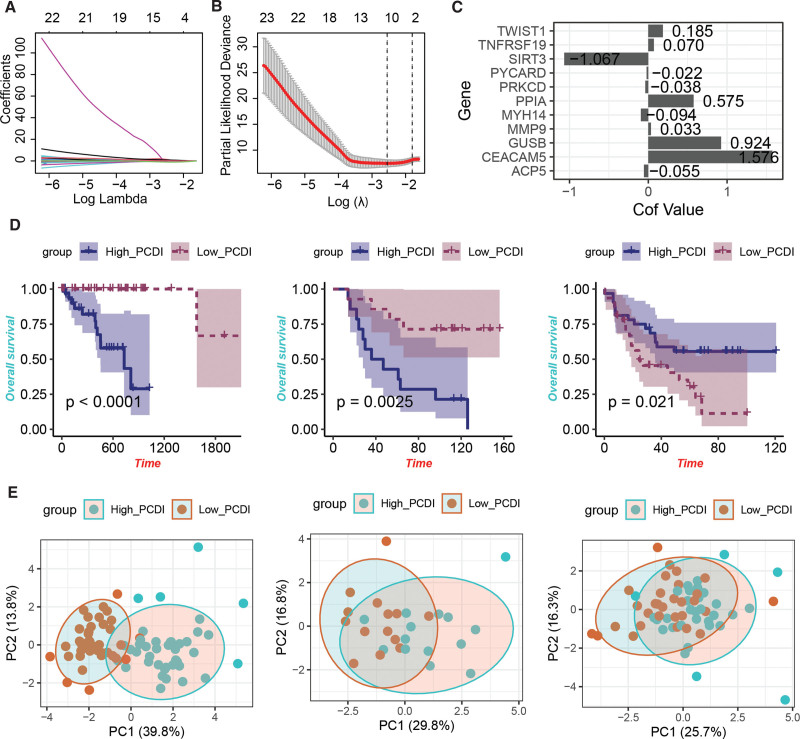
Construction of the PCDI risk signature based on prognostic PCDRGs. (A) and (B) LASSO Cox regression analysis based on 111 prognostic PCDRGs identified 11 risk genes. (C) Coefficients of the 11 PCDI-related genes. (D) Kaplan–Meier survival curves and log-rank test between the High_PCDI and Low_PCDI groups. (E) Principal component analysis based on the 11 PCDRGs. LASSO = Least Absolute Shrinkage and Selection Operator, PCDI = PCDRG-derived index, PCDRGs = programmed cell death-related genes, TCGA = The Cancer Genome Atlas.

To further quantify the risk of poor prognosis in UM patients, we selected 111 prognostic PCDRGs with *P* < .005 from the previous univariate Cox regression analysis and performed Least Absolute Shrinkage and Selection Operator Cox regression analysis (Fig. 2A and B). This approach yielded a risk signature comprising 11 PCDRGs (Fig. 2C), with the PCDI calculated as follows: PCDI = 0.185 × TWIST1 + 0.070 × TNFRSF19 – 1.067 × SIRT3 – 0.022 × PYCARD – 0.038 × PRKCD + 0.575 × PPIA – 0.094 × MYH14 + 0.033 × MMP9 + 0.924 × GUSB + 1.576 × CEACAM5 – 0.055 × ACP5. Patients in the TCGA-UVM, GSE84976, and GSE22138 cohorts were stratified into High_PCDI and Low_PCDI groups based on the median PCDI value, with the High_PCDI group exhibiting significantly worse prognosis compared to the Low_PCDI group (Fig. 2D). PCA based on the signature genes revealed distinct boundaries between the High_PCDI and Low_PCDI groups in the TCGA-UVM and GSE84976 cohorts (Fig. 2E).

### 3.3. Relationship between PCDI and clinicopathological characteristics of UM patients

**Figure 3.**
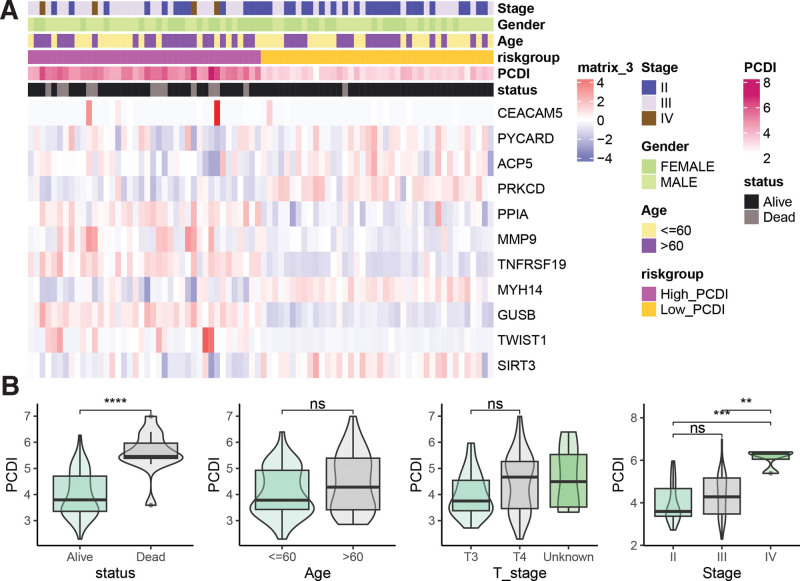
Relationship between PCDI and clinicopathological features of UM patients. (A) Heatmap of PCDI constituent gene expression and annotation of clinicopathological features. (B) Comparison of PCDI among different clinicopathological feature groups. **P < .01, ***P < .001, ****P < .0001. PCDI = PCDRG-derived index, UM = uveal melanoma.

A heatmap was generated based on the normalized gene expression levels to compare the differences in gene expression and clinicopathological features between the High_PCDI and Low_PCDI groups (Fig. 3A). Notably, the Low_PCDI group had fewer deceased cases and a lower proportion of patients with stage III and IV disease compared to the High_PCDI group. Furthermore, deceased UM patients exhibited higher PCDI values compared to alive UM patients. Additionally, stage IV patients had higher PCDI values than stage II and III patients (Fig. 3B).

### 3.4. Relationship between PCDI and somatic mutations in UM patients

**Figure 4.**
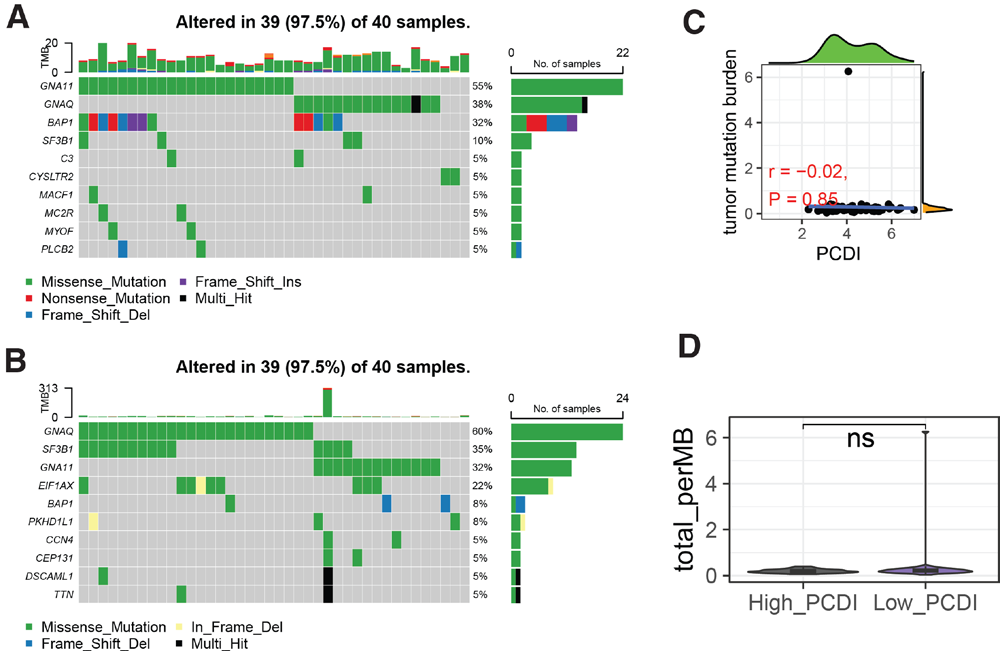
Relationship between PCDI and somatic mutation features of UM patients. (A) Oncoplot of somatic mutations in the High_PCDI group of UM patients. (B) Oncoplot of somatic mutations in the Low_PCDI group of UM patients. (C) Scatter plot of the correlation between PCDI and TMB. (D) Comparison of TMB between the High_PCDI and Low_PCDI groups. ns, not significant, PCDI = PCDRG-derived index, TMB = tumor mutational burden, UM = uveal melanoma.

In the High_PCDI group, the top 10 most frequently mutated genes were GNA11, GNAQ, BAP1, SF3B1, C3, CYSLTR2, MACF1, MC2R, and MYOF (Fig. 4A), whereas in the Low_PCDI group, they were GNAQ, SF3B1, GNA11, EIF1AX, BAP1, PKHD1L1, CCN4, CEP121, DSCAML1, and TTN (Fig. 4B). However, no significant correlation was observed between PCDI and tumor mutational burden (Fig. 4C), and there was no significant difference in tumor mutational burden between the High_PCDI and Low_PCDI groups (Fig. 4D).

### 3.5. Relationship between PCDI and gene expression in UM

**Figure 5.**
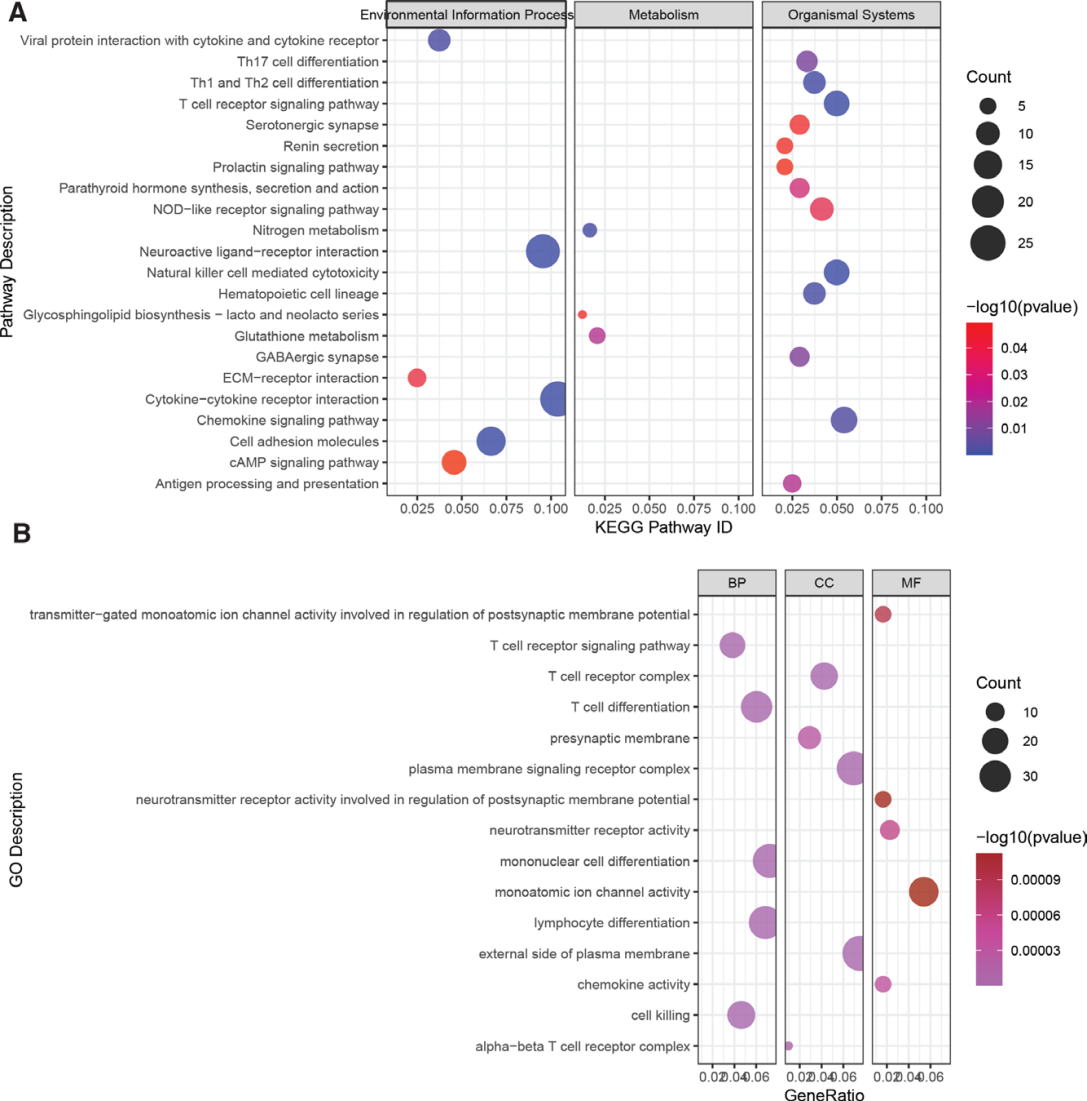
Relationship between PCDI and gene expression regulation in UM patients. (A) KEGG enrichment analysis of differentially expressed genes between the High_PCDI and Low_PCDI groups. (B) GO enrichment analysis of differentially expressed genes between the High_PCDI and Low_PCDI groups. KEGG = Kyoto Encyclopedia of Genes and Genomes, PCDI = PCDRG-derived index, UM = uveal melanoma.

To investigate the relationship between PCDI and gene expression in UM, we identified differentially expressed genes (DEGs) between the High_PCDI and Low_PCDI groups, resulting in 867 DEGs (*P* < .05, |log(fold change)| > 2). Enrichment analysis revealed that these DEGs were associated with pathways related to cytokine signaling, T-cell differentiation, antigen processing and presentation (Fig. 5A). Furthermore, Gene Ontology enrichment analysis demonstrated that these genes were involved in biological processes such as mononuclear cell differentiation, lymphocyte differentiation, cell killing, and T-cell differentiation (Fig. 5B), suggesting differences in the immune status between the High_PCDI and Low_PCDI groups.

### 3.6. Relationship between PCDI and drug sensitivity

**Figure 6.**
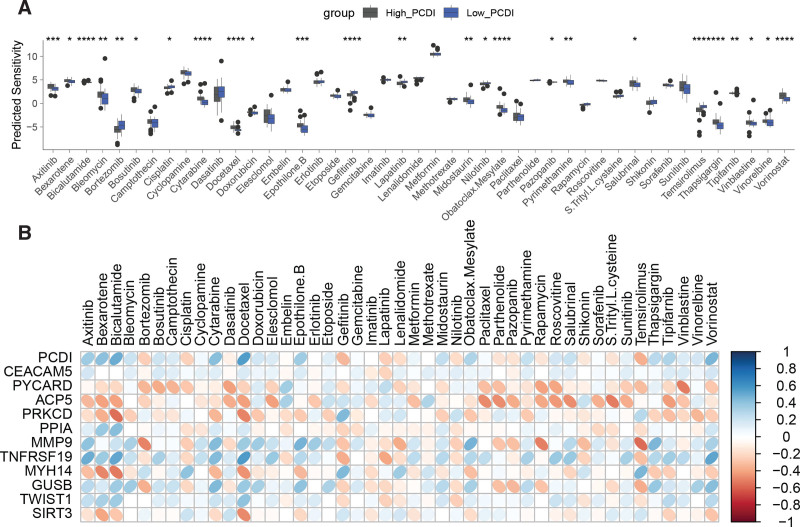
Relationship between PCDI and drug sensitivity of UM patients. (A) Differential analysis of drug sensitivity to 45 drugs between the High_PCDI and Low_PCDI groups. (B) Heatmap of the correlation between PCDI, its constituent genes, and sensitivity to 45 drugs. *P < .05, **P < .01, ***P < .001, ****P < .0001. PCDI = PCDRG-derived index, UM = uveal melanoma.

We evaluated the sensitivity of the TCGA-UVM cohort to 45 drugs using pRRophetic, and the results showed significant differences in drug sensitivity between the High_PCDI and Low_PCDI groups for 25 drugs. The High_PCDI group exhibited lower sensitivity to bortezomib, cisplatin, gefitinib, lapatinib, nilotinib, and temsirolimus, but higher sensitivity to the other 19 drugs (Fig. 6A). Figure 6B illustrates the correlation between PCDI, its constituent genes, and drug sensitivity, where PYCARD, ACP5, PRKCD, MYH14, and SIRT3 exhibited an opposite correlation trend compared to the other genes and PCDI.

### 3.7. Relationship between PCDI and tumor immune microenvironment

**Figure 7.**
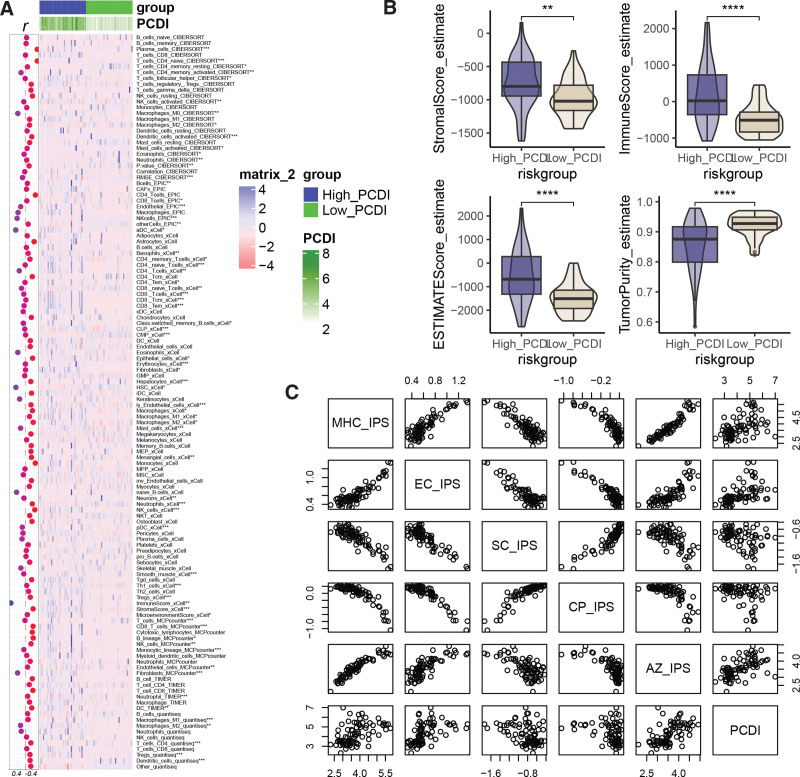
Relationship between PCDI and immune infiltration in UM patients. (A) Differential analysis of immune cell infiltration between the High_PCDI and Low_PCDI groups. (B) Comparison of StromalScore, ImmuneScore, ESTIMATEScore, and Tumor purity between the High_PCDI and Low_PCDI groups. (C) Scatter plots of the correlation between PCDI and MHC_IPS, EC_IPS, SC_IPS, CP_IPS, AZ_IPS. *P < .05, **P < .01, ***P < .001, ****P < .0001. PCDI = PCDRG-derived index, UM = uveal melanoma.

We analyzed the tumor microenvironment in the TCGA-UVM cohort and found that PCDI was negatively correlated with most tumor-infiltrating immune cells. Compared to the High_PCDI group, the Low_PCDI group had higher infiltration levels of CD4 T cells, activated NK cells, macrophages, activated dendritic cells, activated mast cells, and neutrophils (Fig. 7A). Additionally, the High_PCDI group had higher StromalScore, ImmuneScore, ESTIMATEScore, and lower Tumor Purity compared to the Low_PCDI group (Fig. 7B). Figure 7C shows the scatter plot of the correlation between PCDI and IPS, indicating a positive correlation with MHC_IPS, EC_IPS, and AZ_IPS, and a negative correlation with SC_IPS and CP_IPS.

### 3.8. Nomogram for UM patients based on PCDI

**Figure 8.**
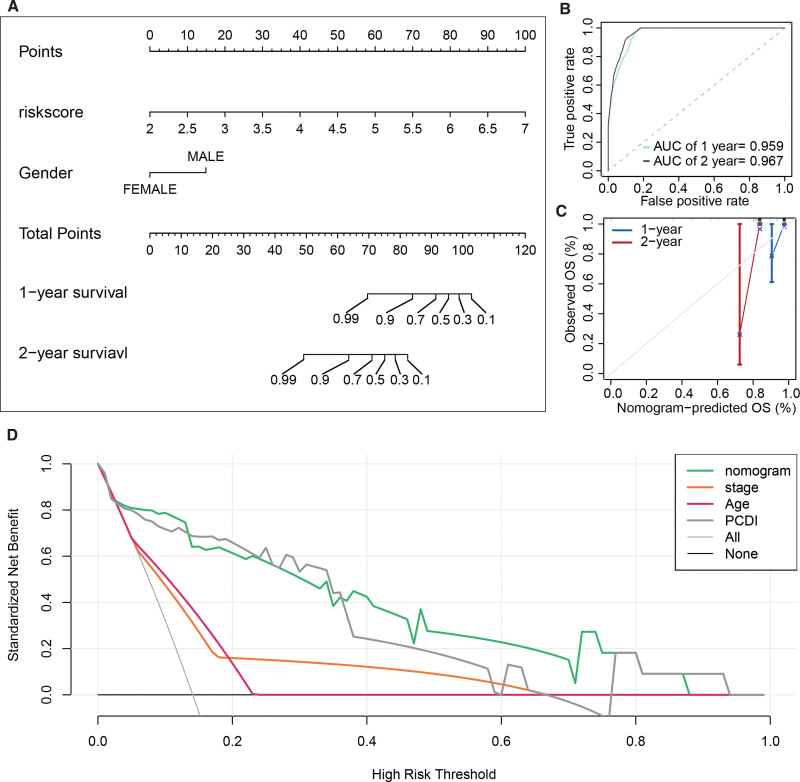
Nomogram construction for the TCGA-UVM cohort based on PCDI. (A) PCDI and Gender, identified as independent prognostic factors, were used to construct a nomogram for predicting the 1- and 2-year overall survival of UM patients. (B) ROC curve analysis of the nomogram for predicting 1-, and 2-year overall survival of UM patients. (C) Calibration curves of the nomogram for predicting 1- and 2-year overall survival of UM patients. (D) Decision curve analysis of the nomogram and other prognostic factors for predicting 1-year overall survival of UM patients. PCDI = PCDRG-derived index, ROC = receiver operating characteristic, TCGA = The Cancer Genome Atlas, UM = uveal melanoma.

In the TCGA-UVM cohort, PCDI, age, and stage were identified as prognostic factors for UM. However, after performing multivariate Cox regression analysis, PCDI and gender emerged as independent prognostic factors (Table 1). Consequently, we constructed a nomogram incorporating PCDI and gender to predict the 1- and 2-year overall survival of UM patients (Fig. 8A). Receiver operating characteristic curve analysis demonstrated the excellent predictive performance of the nomogram, with area under the curve values of 0.959 and 0.967 for predicting 1-, and 2-year overall survival, respectively (Fig. 8B). Figure 8C illustrates the calibration curves of the nomogram for predicting 1- and 2-year overall survival. Notably, compared to other prognostic factors, the nomogram exhibited a higher standardized net benefit in predicting 1-year overall survival for UM patients, indicating its superior prognostic performance over alternative approaches (Fig. 8D).

**Table 1:** Details results of the univariate and multivariate Cox regression analysis.

Characteristics	Univariate Cox				Multivariate Cox			
	HR	Lower.95	Upper.95	*P*	HR	Lower.95	Upper.95	*P*
Risk score	11	3.7	35	2.70E–05	20.41	3.76	110.88	<.001
Age	6	1.3	28	.023	11.65	0.55	244.73	.114
Stage	6.9	1.5	31	.012	2.88	0.27	30.81	.382
Gender	0.3	0.066	1.4	.13	0.1	0.01	0.98	.048
T stage	4	0.84	19	.081	1.87	0.07	48.1	.706

## 4. Discussion

Accumulating evidence suggests that PCDRGs play a crucial role in cancer initiation and progression, and can be utilized for molecular subtyping and prognostic assessment of cancers. However, their value in UM remains unclear. In this study, 1560 PCDRGs were analyzed for their association with UM prognosis, and 369 genes were identified as prognostic PCDRGs. These prognostic PCDRGs were further employed for molecular subtyping and prognostic assessment of UM, ultimately leading to the construction of the PCDI risk signature. These findings highlight the potential value of PCDRGs as prognostic markers and potential therapeutic targets in UM.

The PCDI, comprising 11 PCDRGs, has been validated for its potential utility in prognostic assessment of UM patients, suggesting the potential roles of these genes in UM initiation and progression. Among them, TWIST1 encodes a transcription factor that promotes epithelial-mesenchymal transition, tumor invasion, and metastasis when overexpressed.^[19]^ TNFRSF19 and PYCARD are involved in the regulation of apoptosis and inflammatory responses, respectively, and their dysregulation is closely associated with tumorigenesis and drug resistance.^[20–22]^ Inactivation of SIRT3 can promote tumor metabolic reprogramming and genomic instability.^[23]^ Aberrations in PRKCD and MYH14 can lead to dysregulated cell cycle and enhanced tumor cell migration.^[24,25]^ On the other hand, MMP9 encodes matrix metalloproteinase 9, which participates in extracellular matrix degradation and plays a crucial role in tumor invasion and metastasis.^[26,27]^ Aberrant expression of PPIA, GUSB, and CEACAM5 has also been observed in certain cancers and may be associated with tumor progression and potential tumor markers.^[28–32]^ Additionally, ACP5, encoding acid phosphatase prostate, is involved in bone metabolism, and its dysregulation may be related to bone metastasis.^[33]^ Given the known roles of these genes in cancer, further investigation into their functions and mechanisms in UM is warranted.

Accumulating evidence suggests that the tumor microenvironment plays a pivotal role in cancer initiation and progression, and numerous studies have demonstrated a close association between the infiltration status of immune cells in the tumor microenvironment and patient prognosis. For instance, Clemente et al^[34]^ found that higher levels of CD8 + T-cell and CD20 + B-cell infiltration in melanoma tumor tissues were associated with longer overall survival in patients. Our study results revealed a negative correlation between PCDI and the infiltration of various immune cells, suggesting that PCDI may influence patient prognosis by modulating the tumor immune microenvironment. Moreover, we found that patients in the high PCDI group had higher StromalScore, ImmuneScore, and ESTIMATEScore, but lower tumor purity. This finding is consistent with previous studies showing that higher StromalScore and ImmuneScore are associated with better prognosis.^[35–37]^ Therefore, PCDI may impact the prognosis of UM patients by regulating the stromal and immune cell components within the tumor microenvironment. Notably, PCDI exhibited different correlation patterns with various immune phenotype scores (IPS), which may reflect its differential roles in regulating distinct immune pathways. In recent years, IPS has been widely employed to assess tumor immune status and predict responses to immunotherapy, such as the study by Charoentong et al,^[38]^ who utilized IPS to evaluate the immunogenicity and immune infiltration of different cancers. Thus, investigating the associations between PCDI and different IPS could shed light on its underlying mechanisms in immune regulation.

Lastly, we developed a nomogram for UM based on PCDI to facilitate clinical application. However, this study has several limitations. Firstly, the PCDI derived from retrospective transcriptomic data analysis has not been validated in prospective studies, limiting its clinical utility. Secondly, the functions and mechanisms of the genes constituting PCDI in UM are largely unknown, necessitating further in vitro and in vivo experimental validation. Finally, by constructing the prognostic model solely based on gene expression data, without integrating other multi-omics data (e.g., gene mutations, DNA methylation), the heterogeneity of cancer may not be comprehensively captured.

## 5. Conclusion

In conclusion, this study provides novel insights into the prognostic relevance of PCDRGs in UM and their potential applications in molecular subtyping, prognostic assessment, and personalized treatment strategies. The constructed PCDI risk signature and the developed nomogram hold promise for improving risk stratification and guiding clinical decision-making for UM patients. However, further research is required to validate the clinical utility of PCDI and elucidate the underlying mechanisms of PCDRGs in UM pathogenesis.

## Author contributions

**Conceptualization:** Zibin Liu, Lili Yu, Jian Lai.

**Data curation:** Zibin Liu, Lili Yu, Jian Lai.

**Formal analysis:** Zibin Liu, Lili Yu, Jian Lai.

**Funding acquisition:** Zibin Liu, Rui Zhang.

**Investigation:** Zibin Liu, Lili Yu, Jian Lai.

**Methodology:** Zibin Liu, Lili Yu, Jian Lai.

**Project administration:** Zibin Liu, Lili Yu, Jian Lai.

**Resources:** Zibin Liu, Lili Yu, Jian Lai.

**Software:** Zibin Liu, Lili Yu, Jian Lai.

**Supervision:** Zibin Liu, Lili Yu, Jian Lai.

**Validation:** Zibin Liu, Lili Yu, Jian Lai.

**Visualization:** Zibin Liu, Lili Yu, Jian Lai.

**Writing – original draft:** Zibin Liu, Lili Yu, Jian Lai.

**Writing – review & editing:** Zibin Liu, Lili Yu, Jian Lai, Rui Zhang.

## Supplementary Material




